# RGD-modified hollow mesoporous nanoparticles loaded with cisplatin for antitumor therapy in colon cancer

**DOI:** 10.1186/s12885-026-15923-5

**Published:** 2026-04-11

**Authors:** Xiaohou Xi, Bohao Zhang

**Affiliations:** 1 Department of Oncology, Xi’an International Medical Center Hospital, Xi’an, Shaanxi 710100 People’s Republic of China; 2 Department of Gastroenterology, Jilin Province People´s Hospital, Changchun, Jilin 130021 People’s Republic of China

**Keywords:** Colon cancer, Hollow mesoporous manganese dioxide, Cisplatin, RGD peptide, Nanodrug delivery system, Tumor immune microenvironment

## Abstract

**Objective:**

To develop an Arg-Gly-Asp peptide (RGD)-targeted hollow mesoporous manganese dioxide nanoparticle system loaded with cisplatin (RGD-HMnO₂/CDDP) and evaluate its in vitro antitumor activity, in vivo therapeutic efficacy, and mechanisms in colon cancer.

**Methods:**

Hollow MnO₂ (HMnO₂) was fabricated using SiO₂ nanospheres as a hard template via KMnO₄ oxidative deposition followed by Na₂CO₃ etching. Cisplatin was loaded and RGD peptides were conjugated to obtain RGD-HMnO₂/CDDP. Morphology and physicochemical properties were characterized by TEM, XRD, XPS, DLS, and zeta potential. In CT26 cells, cytotoxicity (CCK-8), invasion/migration (Transwell), reactive oxygen species (ROS, DCFH-DA), mitochondrial membrane potential (JC-1), DNA damage (γ-H2AX), apoptosis (Western blot; Annexin V-FITC/PI) were assessed. In tumor-bearing mice, antitumor efficacy, serum cytokines (TNF-α, IFN-γ, IL-6, IL-2, IL-10), and hematological indices were evaluated.

**Results:**

RGD-HMnO₂/CDDP showed markedly enhanced cytotoxicity versus free cisplatin and non-targeted HMnO₂/CDDP, and significantly inhibited invasion and migration. Mechanistically, it increased intracellular ROS, induced mitochondrial depolarization, elevated γ-H2AX, and activated apoptosis signaling, resulting in higher apoptotic rates. In vivo, RGD-HMnO₂/CDDP achieved superior tumor growth inhibition, modulated inflammatory/immune cytokines, and maintained favorable hematological profiles, indicating good immunomodulation and biocompatibility.

**Conclusion:**

RGD-HMnO₂/CDDP enhances cisplatin efficacy against colon cancer by promoting ROS-driven mitochondrial dysfunction and DNA damage–associated apoptosis, with potential improvement of the tumor immune microenvironment and without compromising systemic safety.

**Supplementary Information:**

The online version contains supplementary material available at 10.1186/s12885-026-15923-5.

## Introduction

Colorectal cancer (CRC) remains one of the leading contributors to the global cancer burden, and both GLOBOCAN 2020 and recent annual statistics indicate persistently high incidence and mortality rates [1, 2]. CRC is characterized by substantial molecular subtypes and clinical heterogeneity; locally advanced disease, distant metastasis, and postoperative recurrence are major determinants of poor prognosis [3]. For recurrent or metastatic CRC, ESMO consensus recommendations and NCCN guidelines highlight combination chemotherapy regimens built on fluoropyrimidines and, when appropriate, the addition of anti-angiogenic or anti-EGFR targeted agents guided by molecular stratification [4, 5]. Immune checkpoint inhibition confers marked survival benefits in MSI-H/dMMR disease, yet this subgroup represents a minority, and most patients still rely on chemotherapy-dominant systemic treatment [6]. Therefore, improving effective drug exposure and overcoming resistance without increasing systemic toxicity remains an important unmet need in CRC therapy.

Platinum drugs are canonical DNA-damaging chemotherapeutics that form DNA adducts and trigger replication arrest and cell-death signaling [7, 8]. However, cisplatin commonly encounters a limited therapeutic window in solid tumors: non-specific biodistribution leads to insufficient intratumoral concentrations, and tumor cells can acquire resistance through enhanced DNA repair, altered drug uptake/efflux, glutathione-associated detoxification, and activation of anti-apoptotic pathways [9]. Moreover, multi-organ toxicities-particularly nephrotoxicity-restrict dose intensity and treatment duration [10, 11]. These challenges suggest that simply escalating dose is unlikely to yield sustained benefit; instead, delivery optimization and rational sensitization strategies are more clinically actionable.

A further structural barrier in solid tumors is tissue-level delivery limitation. Dense stroma, heterogeneous perfusion, and elevated interstitial pressure compromise drug and nanoparticle penetration into tumor cores, creating a delivery bottleneck of inadequate intratumoral exposure [12]. Nanomedicine can improve pharmacokinetics and provides an engineering interface for targeting and stimuli-responsive release [13, 14]. Nonetheless, systematic analyses indicate that overall tumor delivery efficiency is strongly constrained by biological barriers and tumor architecture, and passive accumulation alone often fails to translate reliably into therapeutic advantage [15, 16]. Accordingly, a design paradigm that integrates barrier crossing, enhanced tissue penetration, improved cellular internalization, and microenvironmental adaptation is critical for effective nanotherapeutics [17, 18]. 

The tumor microenvironment (TME) is typically hypoxic, mildly acidic, and highly reducing, which can reduce chemosensitivity and limit effector immune-cell infiltration [19]. Accumulating evidence highlights that the TME is highly heterogeneous across solid tumors, spanning immune-active (“hot”) and immunosuppressive (“cold”) states characterized by distinct immune-cell compositions and cytokine signaling programs [20]. Such immune-contexture differences are closely associated with tumor progression, therapeutic responsiveness, and clinical outcomes. Recent integrative single-cell and machine-learning–based studies further demonstrate that cytokine networks and immune-regulatory pathways serve as key indicators of TIME status, while tumor metabolic reprogramming can actively shape immune suppression and immune resistance [21, 22]. Hypoxia-driven signaling (e.g., HIF-1) is tightly linked to metabolic reprogramming, invasion/metastasis, and therapeutic resistance, making this axis an attractive entry point for sensitization [23]. Thus, microenvironment-responsive nanoplatforms that locally alleviate hypoxia and redox stress while releasing cytotoxic payloads are biologically rational. From a delivery standpoint, integrin-mediated active targeting offers a feasible route to enhance tumor-specific delivery. Arg-Gly-Asp peptide (RGD)-binding integrins (e.g., αvβ3) participate in tumor cell adhesion and migration, angiogenesis, and metastasis, and represent important targets for cancer diagnosis and therapy [24, 25]. To address the limitations of single-ligand targeting under tumor heterogeneity, dual-targeting combinations incorporating RGD and other receptors/therapeutic modules have been explored to improve cellular uptake and specificity [26], and RGD-based self-assembling nanodrug strategies have been developed to enable high loading and multifunctional synergy [27]. Incorporating RGD into platinum delivery systems may improve tumor retention and cellular internalization, thereby providing delivery-based support for enhanced exposure and reduced off-target toxicity. Manganese dioxide (MnO₂) can undergo microenvironment-triggered degradation under acidic and high-glutathione (GSH) conditions, release Mn2+, and participate in H_2_O_2_-related reactions, offering the potential to couple microenvironment adaptation with local chemical modulation. Recent reviews emphasize programmable advantages of manganese oxide nanomaterials in pH-responsive release, GSH depletion, hypoxia relief, and synergistic chemo-/immunotherapy [28]. Functionalized MnO₂ nanosheets and MnO₂-based therapeutic systems have been widely used to construct combinatorial strategies integrating oxygenation, redox modulation, and sensitization [29, 30]. In parallel, catalytic nanomaterials with nanozyme-like properties are emerging in oncology, as peroxidase-like activities can drive reactive oxygen species (ROS) cascades and promote oxidative stress–associated cell death [31, 32]. 

Mechanistically, the mode of cellular stress and death not only dictates tumor-cell clearance efficiency but also shapes downstream immune recognition and treatment response. Immunogenic cell stress and death has been proposed as a key hub connecting intracellular stress to antitumor immune activation [33], whereas therapy-induced cell death can elicit bidirectional effects-both immune-stimulatory and tissue-repair/immune-suppressive-highlighting the need to balance effective killing with controllable inflammation [34]. At the molecular level, mitochondria are central regulators of multiple death programs [35], and mitochondrial outer membrane permeabilization regulated by the BCL-2 family is a core event in intrinsic apoptosis and an actionable drug target [36]. Recent NCCD updates further emphasize that cell-death phenotypes should be described in a mechanism-oriented manner while considering their functional consequences in disease and therapy [37, 38]. 

Based on this rationale, we constructed an RGD-modified hollow mesoporous MnO₂ nanoplatform loaded with cisplatin, aiming to enhance colon cancer treatment via a coordinated cascade of active tumor targeting, microenvironment-responsive modulation, and oxidative-stress amplification. We evaluated its in vitro antitumor activity and key death pathways, and validated therapeutic potential and safety trends in a CRC tumor-bearing mouse model. Collectively, this work provides experimental evidence and design cues for nanomedicine-enabled and precision-oriented optimization of platinum chemotherapy.

## Materials and methods

### Synthesis of hollow mesoporous manganese dioxide (HMnO_**2**_)

Firstly, a reaction solution for the hydrolysis of tetraethyl orthosilicate (TEOS) was prepared by mixing 25 mL of anhydrous ethanol, 2 mL of ultrapure water, and 3 mL of ammonia water, followed by stirring to ensure homogeneity. The solution was maintained at a constant temperature of 45 °C in a thermostatic oil bath for 10 min. Then, 3 mL of TEOS was rapidly added to the stirring reaction mixture, and the reaction continued overnight, yielding silica nanospheres. Secondly, hollow mesoporous manganese dioxide was synthesized by using the as-prepared silica spheres as a template. The silica spheres were subjected to ultrasonic treatment, and a solution containing 150 mg of potassium permanganate (KMnO_4_) in 50 mL of solvent was added dropwise under continuous sonication for 6 h. After ultrasonication, the mixture was centrifuged at 14,800 rpm and washed three times with water, resulting in dark brown SiO_2_@MnO_2_ nanospheres. Finally, the obtained SiO_2_@MnO_2_ nanospheres were dispersed in 20 mL of 2 mol/L Na_2_CO_3_ solution and stirred at 60 °C overnight. The Na_2_CO_3_ solution selectively etched the silica from the SiO_2_@MnO_2_ structure, resulting in hollow mesoporous manganese dioxide nanospheres (HMnO_2_). The following morning, the product was centrifuged at 14,800 rpm and washed three times to remove sodium silicate, yielding the desired hollow mesoporous manganese dioxide nanospheres (HMnO_2_).

### Synthesis of cisplatin-loaded hollow mesoporous manganese dioxide (HMnO_**2**_-Cisplatin)

100 mg of hollow mesoporous manganese dioxide was dispersed in a DMSO solution and subjected to ultrasonic dispersion. 100 mg of cisplatin was added, and the mixture was incubated overnight at 37 °C on a shaking incubator. After incubation, the mixture was centrifuged at 11,000 rpm and washed three times with pure water to obtain the cisplatin-loaded hollow mesoporous manganese dioxide (HMnO_2_-Cisplatin).

### Synthesis of RGD-modified cisplatin-loaded hollow mesoporous manganese dioxide (HMnO_**2**_-Cisplatin-RGD)

To the 2 mg/mL solution of HMnO_2_, 10 mL of 5 mg/mL poly(allylamine hydrochloride) (PAH) was added dropwise, and the mixture was sonicated for 2 h under continuous stirring. The resulting nanoparticles were centrifuged and washed three times to remove excess polymer molecules. Subsequently, the nanoparticles were dispersed in 10 mL of 5 mg/mL poly (acrylic acid) (PAA) solution and sonicated for 2 h. The mixture was centrifuged at 14,800 rpm to remove the supernatant, and the particles were washed three times and dispersed in 5 mL of deionized water, yielding the polymer-modified hollow mesoporous manganese dioxide (HMnO_2_-PP). Next, the cisplatin-loaded nanoparticles (HMnO_2_-PP) were dispersed in MES buffer solution, and 100 mg of EDC and 120 mg of NHS were added. The mixture was incubated on a shaking incubator for 0.5 h. After adjusting the pH to a weakly alkaline condition using a BB buffer, 10 mg of RGD was added, and the reaction was allowed to proceed overnight at 37 °C, resulting in RGD-modified cisplatin-loaded hollow mesoporous manganese dioxide (HMnO_2_-Cisplatin-RGD).

### Physicochemical characterization

The morphology and hollow structure of the samples were observed using Transmission Electron Microscopy (TEM). The crystallographic structure was analyzed by X-ray Diffraction (XRD) using a Rigaku instrument (Japan). The elemental composition and oxidation states were determined by X-ray Photoelectron Spectroscopy (XPS) (Thermo Fisher Scientific, USA). Dynamic Light Scattering (DLS) was employed to measure the hydrodynamic diameter and particle size distribution. The surface charge variations were assessed through Zeta potential analysis using a Malvern Instruments (UK) device.

### Cell culture

Mouse CRC cells (CT26, catalog number: CRL-2638) was obtained from the American Type Culture Collections and authenticated by the institution prior to experimental use. CT26 cells were cultured under standard conditions and divided into five groups: Control (G1), HMnO₂ carrier (G2), Cisplatin (CDDP) (G3), HMnO₂/CDDP (G4), and RGD-HMnO₂/CDDP (G5). All cells were authenticated and free of mycoplasma.

### CCK-8 cytotoxicity assay

The cytotoxicity was determined using the CCK-8 (Beyotime, China) assay. Briefly, CT26 cells were seeded in 96-well plate at a density of 1.5 × 10^4^ cells and incubated for 24 h to allow cell attachment. Then, the cells were cultured with different concentrations for 24, hours. CCK-8 test solution was added to each well of the plate (the volume of the CCK-8 test solution in each well is ^1^/_10_ of the total volume) and incubated for another 3 h. After selective reduction by viable cells, the absorption intensity of each well at 450 nm was measured. Percentage of the viability was normalized according to the untreated cells.

### Intracellular ROS measurement (DCFH-DA)

Intracellular ROS was examined using the oxidation-sensitive dye DCFH-DA(Beyotime, China). Briefly, CT26 cells were plated in 24 -plates and exposed to the indicated treatments (G1–G5) for 24 h. Cells were then incubated with DCFH-DA working solution at 37 °C for 20 min in the dark. After three gentle rinses with PBS to remove unbound probe, fluorescence signals were visualized using a fluorescence microscope.

### Assessment of mitochondrial membrane potential by JC-1

Mitochondrial membrane potential (ΔΨm) was evaluated using JC-1 staining (Beyotime, China). Cells were seeded in 24-well plates (4 × 10⁴ cells per well) and treated for 24 h. Following treatment, cells were washed with PBS and incubated with JC-1 staining solution for 30 min at 37 °C protected from light. After removal of the staining solution, cells were washed twice to eliminate residual dye and maintained in serum-free medium prior to imaging. The green fluorescence (JC-1 monomers) and red fluorescence (JC-1 aggregates) were captured using an inverted fluorescence microscope.

### Transwell invasion assay

Invasive capacity was determined using a Matrigel (Basement Membrane Matrix)-coated Transwell system. Cells were serum-deprived for 12 h, collected, and resuspended in serum-free medium. A total of 2 × 10⁴ cells were added to the upper compartment in 100 µL, whereas the lower chamber was filled with complete medium containing 20% FBS as a chemoattractant. After 24 h incubation at 37 °C with 5% CO₂, cells remaining on the upper surface were removed, and invaded cells on the underside were fixed with 4% paraformaldehyde and stained with 0.5% crystal violet for 30 min. Images were obtained under a brightfield microscope.

### γ-H2AX immunofluorescence staining for DNA double-strand breaks

DNA double-strand breaks were evaluated by γ-H2AX immunofluorescence staining. After the treatments, culture medium was removed and cells were rinsed once with PBS. Cells were immediately fixed with 4% PFA for 5–15 min at room temperature. Following fixation, samples were washed three times with wash buffer (3–5 min each), ensuring complete removal of residual liquid while preventing the cell surface from drying. Cells were then incubated with a blocking solution for 10–20 min at room temperature, followed by incubation with a rabbit anti-γ-H2AX primary antibody (cat. no. 9718, Cell Signaling Technology, Danvers, Massachusetts) diluted in antibody diluent for 1 h at room temperature or overnight at 4 °C. After primary incubation, samples were washed three times (5–10 min per wash) and subsequently incubated with an Alexa Fluor 488-conjugated anti-rabbit secondary antibody for 1 h at room temperature in the dark. Excess secondary antibody was removed by two additional washes (5–10 min each), and nuclei were counterstained with DAPI for approximately 10 min at room temperature. Samples were washed three times (3–5 min each) prior to imaging.

### Cellular uptake study

The cellular uptake study was performed using fluorescent DIO nanoparticles. CT26 cells were seeded at a density of 1 × 10^5^ cells in a 12-well plate and incubated for 24 h. Cells were exposed with 5 µg/ml of DIO loaded nanoparticles (in media) and incubated for 24 h. Cells were washed twice with PBS and fixed with 4% paraformaldehyde solution for 15 min. Cells were then viewed under confocal laser scanning microscopy.

### Flow cytometric analysis of apoptosis (Annexin V-FITC/PI)

Apoptosis was quantified by Annexin V-FITC/PI double staining followed by flow cytometry. After the treatments, cells were harvested, rinsed twice with ice-cold PBS, and pelleted by centrifugation (1000 rpm, 5 min). The cell pellet was resuspended in 1× binding buffer, and aliquots (100 µL) were incubated with Annexin V-FITC (5 µL) for 15 min at room temperature in the dark; PI (5 µL) was subsequently added and incubated for an additional 15 min. Samples were analyzed on a FACS Calibur flow cytometer (BD Biosciences).

### Western blot

Western blotting was used to determine the protein levels of Bax, Bcl-2, cytochrome c, caspase-9 and caspase-3. Cells were lysed on ice in lysis buffer (1×PBS, 1% NP-40, 0.5% sodium deoxycholate, 0.1% SDS, 10 µg/mL PMSF) and centrifuged at 12,000 rpm for 10 min at 4 °C. The supernatants were collected, and protein concentrations were measured using a Bio-Rad protein assay. Equal amounts of protein were separated by 10% SDS-PAGE and transferred to PVDF membranes. After blocking with 5% nonfat milk in PBST for 1 h at room temperature, membranes were incubated with primary antibodies against Bax, Bcl-2, cytochrome c, cleaved caspase-3, cleaved caspase-9, and β-actin (Santa Cruz Biotechnology) overnight at 4 °C, followed by HRP-conjugated secondary antibodies for 1 h at room temperature. Signals were developed and band intensities were quantified using ImageJ.

### Animals

All animal procedures were conducted in accordance with institutional animal care procedures and NIH guidelines and were approved by the Xi’an International Medical Center Hospital Animal Care Committee. Female BALB/c mice (6–8 weeks old, 18–22 g, SPF grade; Beijing Vital River Laboratory Animal Technology Co., Ltd.) were acclimated for one week prior to tumor establishment. CT26 cells at the exponential growth phase were harvested and resuspended at 2 × 10^7^ cells/mL. The cell suspension was mixed with PBS and Matrigel (1:1, v/v), and 100 µL of the mixture (2 × 10^6^ cells) was subcutaneously inoculated into the right axilla of each mouse. The mice were anesthetized with 2% isoflurane (Shanghai Abbott Pharmaceutical Co. Ltd.) during imaging and then humanely euthanized via CO_2_ inhalation at the experimental endpoint. Tumor volume was calculated as V = (length × width^2^)/2, and mice were randomized for subsequent experiments when tumor volumes reached approximately 100–200 mm^3^.

For in vivo fluorescence imaging, tumor-bearing mice were randomly assigned to a targeting group (ICG-RGD-HMnO₂/CDDP) or a non-targeting group (ICG-HMnO₂/CDDP) (*n* = 3 per group). Formulations containing an equivalent dose of ICG were administered via tail vein injection. Whole-body fluorescence imaging was performed at 0, 2, 6, 12, 24, and 36 h post-injection using identical acquisition settings.

For in vivo therapy and safety evaluation, tumor-bearing mice were randomized into five groups (*n* = 5 per group): PBS, HMnO₂, CDDP, HMnO₂/CDDP, and RGD-HMnO₂/CDDP. All treatments were administered intravenously via the tail vein at an equivalent cisplatin dose (2.5 mg/kg as CDDP, or an experimentally equivalent dose) every 3 days for a total of four injections. Tumor volume and body weight were monitored every 2–3 days throughout the treatment period. After the final administration, serum samples were collected and cytokine levels (TNF-α, IFN-γ, IL-6, IL-2, and IL-10) were determined using ELISA kits. Peripheral blood was collected in parallel for routine hematological analysis, including white blood cells (WBC), red blood cells (RBC), and platelets (PLT), to assess systemic safety.

### Statistical analysis

All quantitative data are presented as mean ± standard deviation (SD). Comparisons between two groups were performed using a two-tailed Student’s *t*-test. Comparisons among multiple groups were conducted by one-way analysis of variance (one-way ANOVA) followed by Tukey’s post hoc test. A *P* value < 0.05 was considered statistically significant.

## Results

### Construction of HMnO₂ nanocarriers and characterization of RGD surface functionalization

**Fig. 1:**
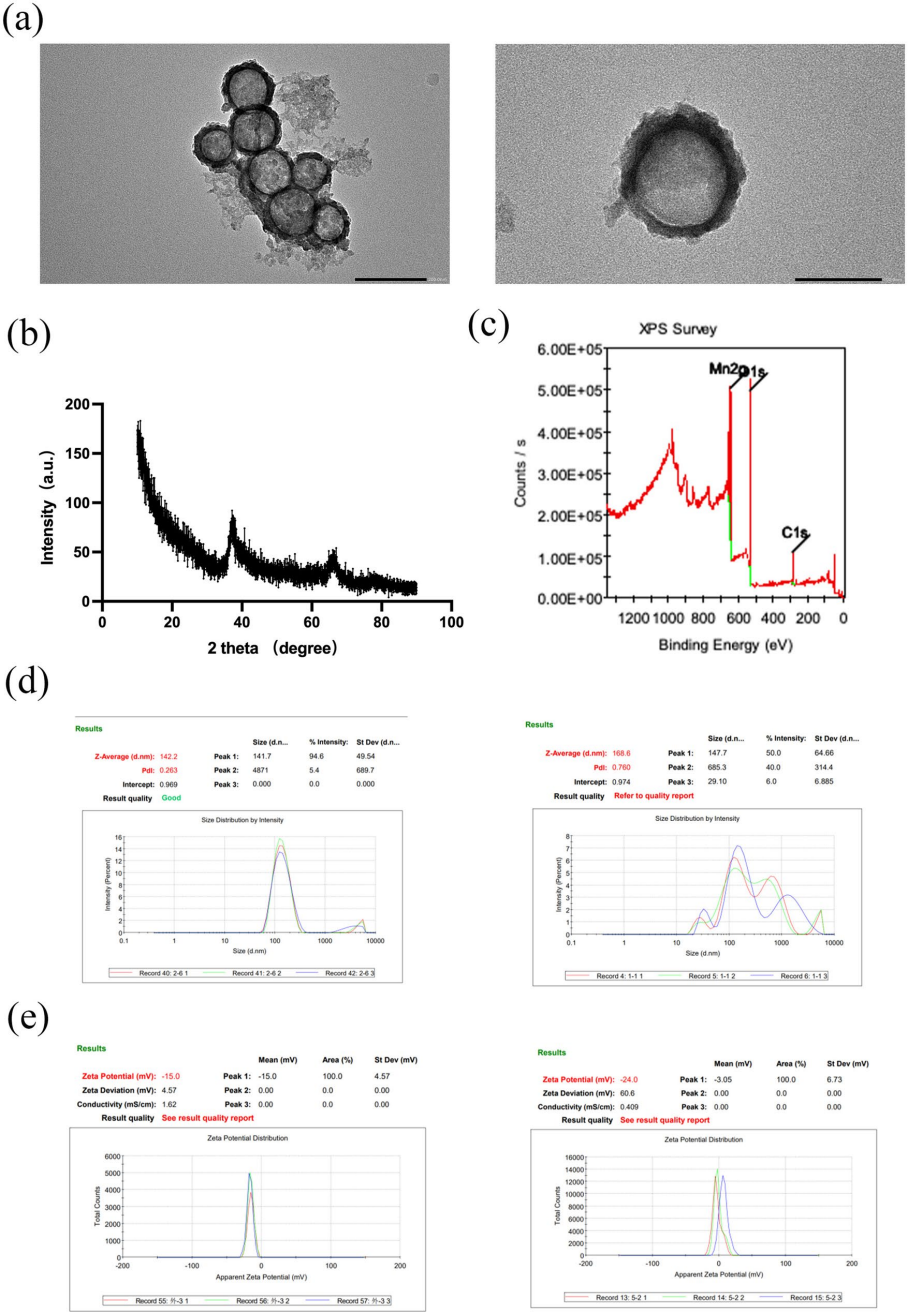
Morphology and physicochemical characterization of HMnO₂ and RGD-modified nanoparticles. **a** TEM images of HMnO₂ nanoparticles showing a uniform spherical morphology and a hollow mesoporous structure (scale bars as indicated). **b** XRD pattern of HMnO₂. **c** XPS survey spectrum of HMnO₂. **d** Hydrodynamic size distributions and Z-average measured by DLS (left: HMnO₂; right: RGD-HMnO₂). **e** Zeta potential distributions (left: HMnO₂; right: RGD-HMnO₂)

TEM revealed that HMnO₂ nanoparticles displayed a regular, near-spherical morphology with a distinct internal cavity and well-defined shell structure, consistent with a hollow mesoporous architecture (Fig. 1a), indicating ample loading capacity and a modifiable interface. XRD patterns showed predominantly broad diffuse peaks (Fig. 1b), consistent with mesoporous/low-crystallinity MnO₂ materials. XPS survey spectra identified characteristic Mn and O signals along with a C 1s peak (Fig. 1c), supporting MnO₂ as the major component with carbon-containing surface species. Colloidal characterization indicated that RGD modification increased the hydrodynamic Z-average from 142.2 nm to 168.6 nm (Fig. 1d), in line with an increased hydration layer and interfacial changes after ligand grafting. Meanwhile, the zeta potential shifted from − 15.0 mV to − 3.05 mV (Fig. 1e), suggesting a marked change in surface charge environment. Together, these data confirm successful RGD functionalization and interfacial remodeling, which may influence subsequent interactions with cell membranes and cellular uptake behavior.

### RGD-HMnO₂/CDDP enhances dose-dependent cytotoxicity and suppresses invasive migration of colon cancer cells

**Fig. 2:**
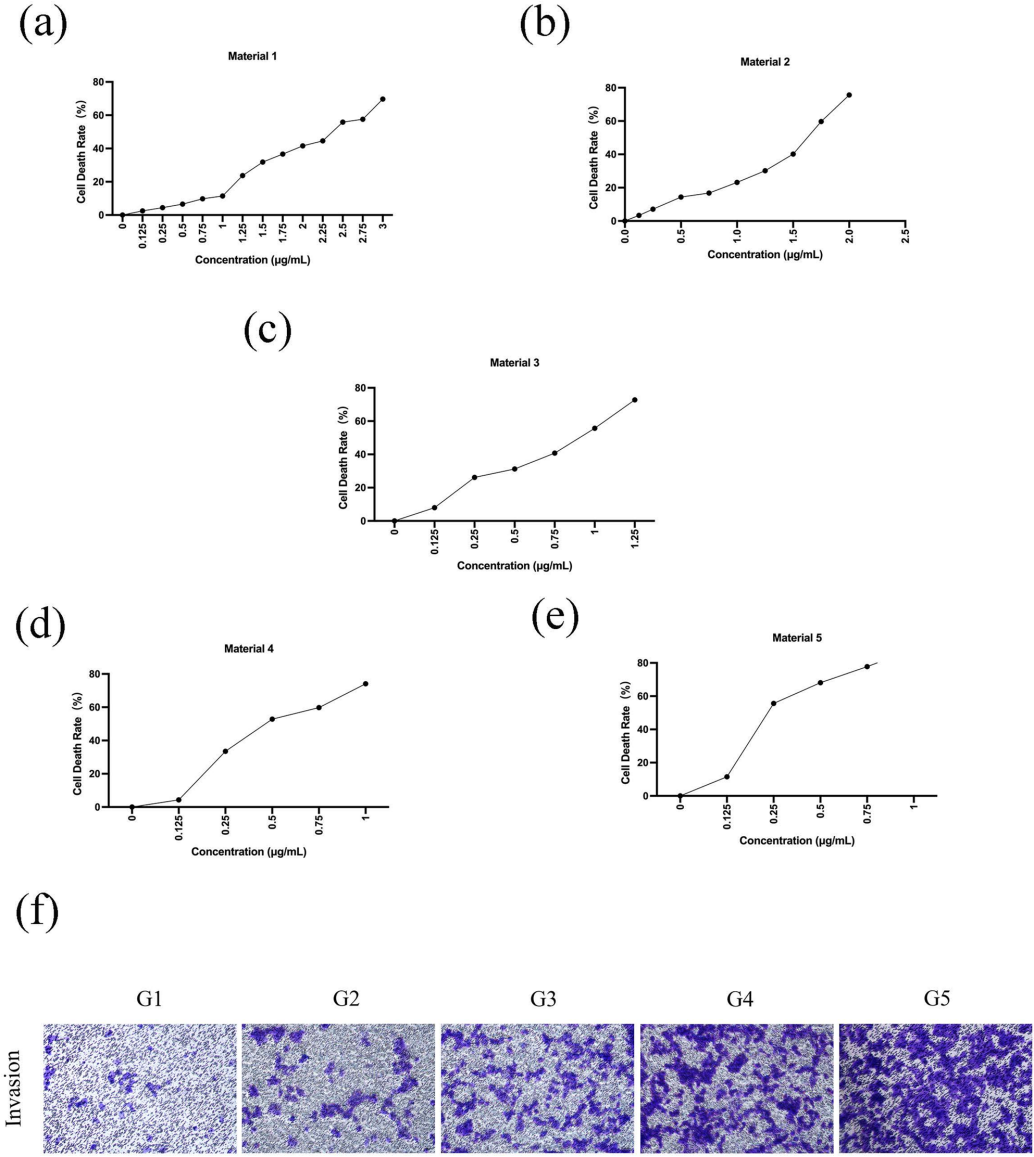
Effects of RGD-HMnO₂/CDDP on CT26 cell viability and invasion. **a**–**e** CCK-8 assay-derived cell death rate (converted from cell viability) of CT26 cells treated with increasing concentrations of each formulation. The x-axis denotes the mass concentration (µg/mL) of the formulation added to the culture (HMnO₂ or drug-loaded nanoparticles); the control group received vehicle only. **f** Representative images of the Transwell invasion assay stained with crystal violet, showing invaded cells after different treatments

In CT26 murine colon carcinoma cells, CCK-8 assays showed a dose-dependent inhibition of cell viability across all treatment groups as formulation concentration increased (Fig. 2a–e). At equivalent cisplatin concentrations, RGD-HMnO₂/CDDP (G5) produced the most pronounced inhibitory effect, suggesting that RGD-mediated targeting and HMnO₂-based delivery synergistically enhanced effective cytotoxic exposure in CT26 cells. Transwell invasion assays further showed an evident reduction in the number of cells crossing the membrane after RGD-HMnO₂/CDDP treatment (Fig. 2f), indicating that, beyond inhibiting proliferation, the targeted delivery system also attenuated invasive and migratory potential.

### RGD-HMnO₂/CDDP induces ros accumulation and mitochondrial membrane potential depolarization in CT26 Cells

**Fig. 3:**
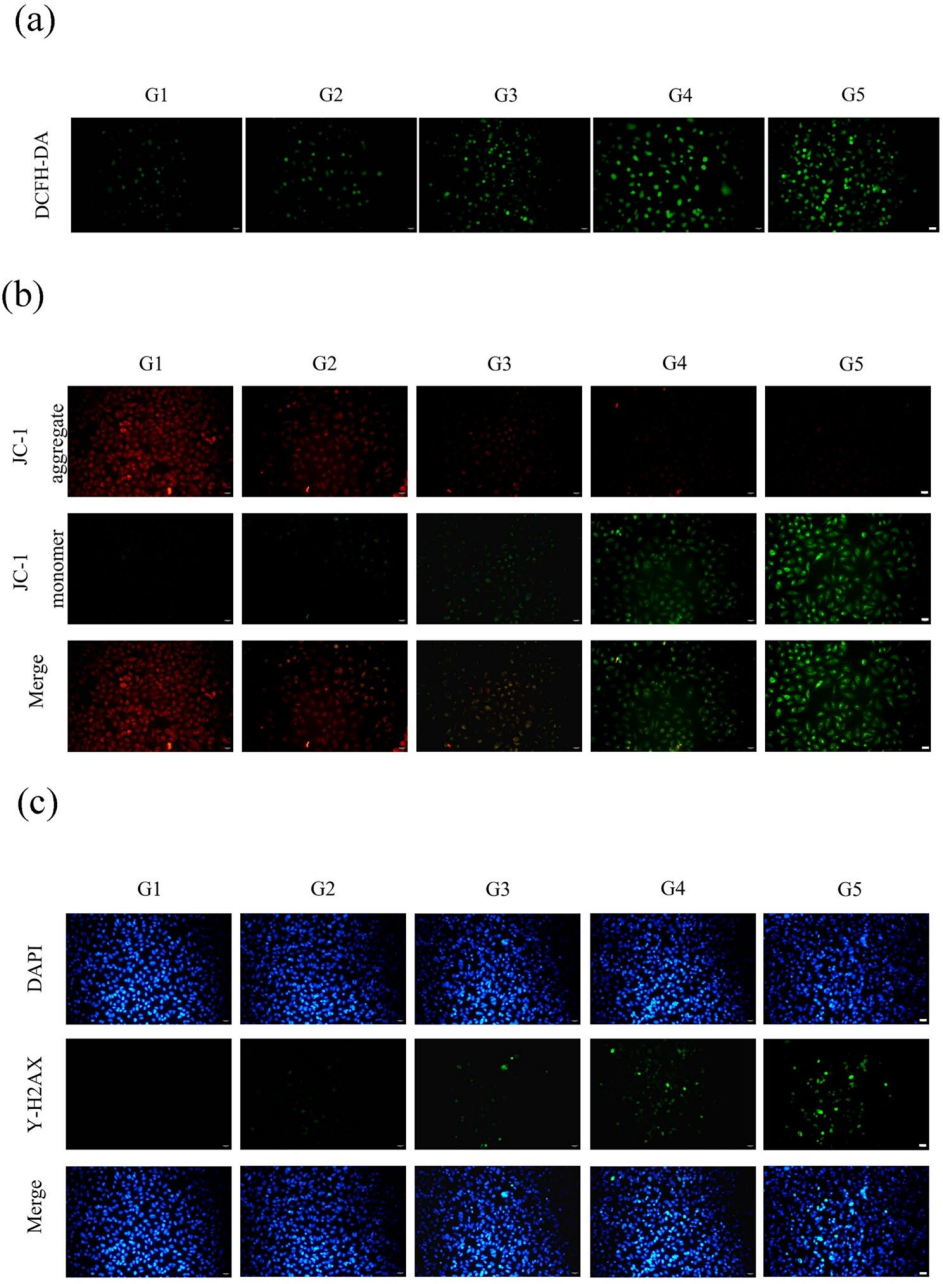
Intracellular ROS, mitochondrial membrane potential, and DNA damage in CT26 cells following nanoparticle treatments. **a** Representative fluorescence images of intracellular ROS detected by the DCFH-DA probe (green fluorescence intensity reflects ROS levels). **b** JC-1 staining to assess ΔΨm: JC-1 aggregates (red) indicate higher ΔΨm, whereas JC-1 monomers (green) indicate reduced ΔΨm; merged images are shown. **c** Immunofluorescence staining of γ-H2AX (green) with DAPI nuclear counterstaining (blue) to indicate DNA double-strand break–associated damage; merged images are shown. Scale bars: 50 µm

In CT26 cells, intracellular reactive oxygen species (ROS) levels were assessed using the DCFH-DA probe. Compared with the control group, all treatment groups showed varying degrees of enhanced green fluorescence, indicating increased ROS generation; the RGD-HMnO_2_/CDDP (G5) group displayed the most pronounced fluorescence, suggesting that the targeted delivery system more effectively promoted oxidative-stress accumulation (Fig. 3a). Mitochondrial membrane potential (ΔΨm) was further evaluated by JC-1 staining. The control group exhibited predominantly red aggregate fluorescence (stable ΔΨm), whereas the treatment groups showed a gradual loss of red signal accompanied by an increase in green monomer fluorescence, indicating ΔΨm depolarization; the G5 group showed the most evident red-to-green shift, implying the strongest impairment of mitochondrial function (Fig. 3b). Meanwhile, γ-H2AX immunofluorescence staining revealed only sparse or nearly undetectable green γ-H2AX signals in the control and carrier groups, whereas drug- and nanoformulation-treated cells exhibited a marked increase in nuclear γ-H2AX-positive foci, consistent with aggravated DNA damage (particularly DNA double-strand break–associated injury); the G5 group presented the strongest γ-H2AX signal and the highest density of positive foci, indicating that RGD-modified HMnO_2_/CDDP more efficiently triggered the DNA damage response (Fig. 3c). Quantitative analysis further corroborated these findings (Figure S1). Taken together, the coordinated increases in ROS, loss of ΔΨm, and elevated γ-H2AX suggest that RGD-HMnO_2_/CDDP markedly amplifies oxidative stress, induces mitochondrial dysfunction, and exacerbates DNA damage, thereby providing mechanistic support for subsequent activation of mitochondria-associated cell death/apoptotic processes.

### RGD improves cellular uptake and potentiates mitochondria-mediated apoptotic signaling

**Fig. 4:**
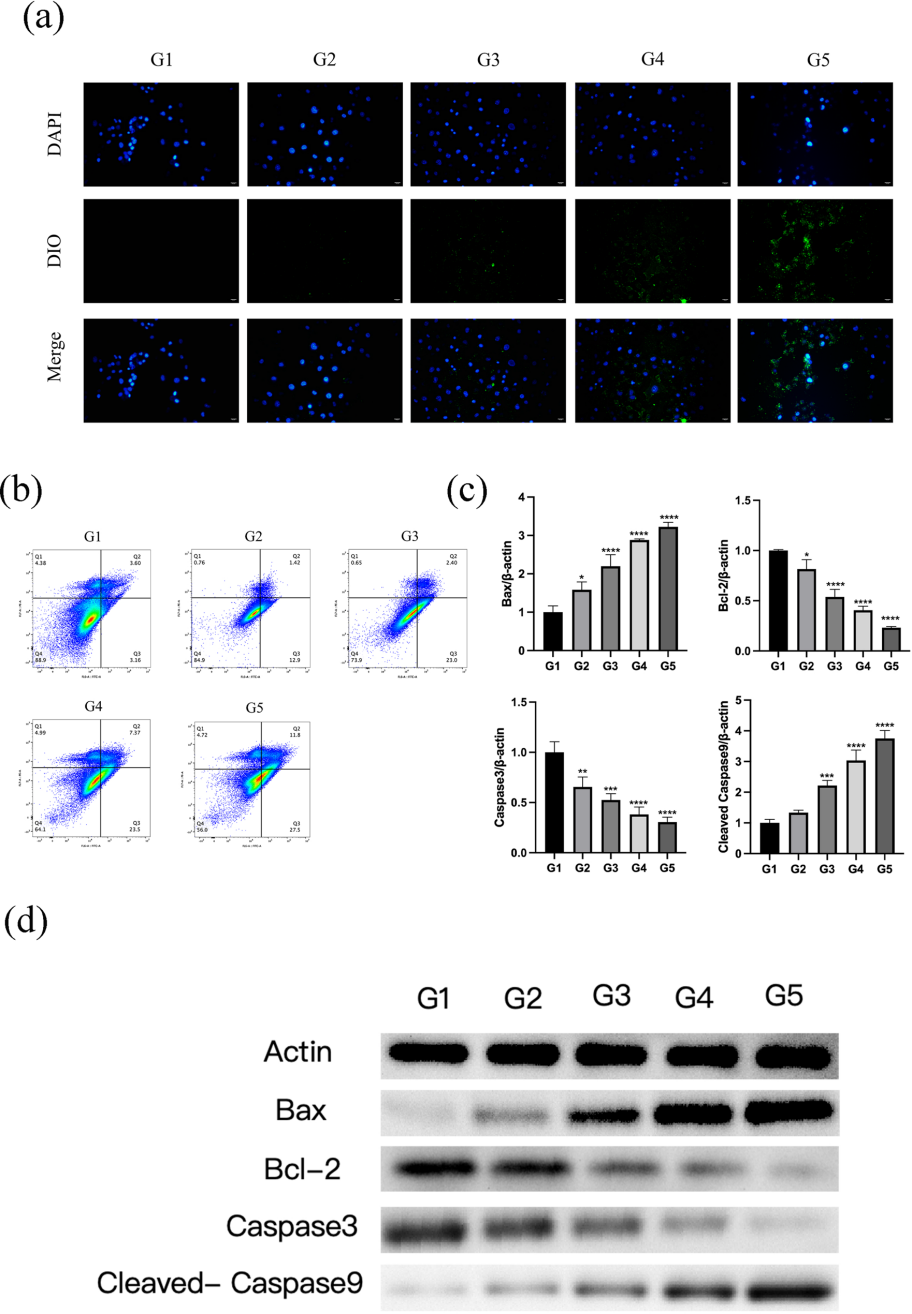
Cellular uptake and apoptosis-related responses of CT26 cells after exposure to RGD-modified nanoparticle formulations. **a** Fluorescence images of cellular uptake of DiO-labeled formulations: nuclei were counterstained with DAPI (blue), and DiO signals (green) indicate internalized formulations; merged images are shown. Scale bars: 50 µm. **b** Representative flow cytometry dot plots of Annexin V-FITC/PI staining for apoptosis. **c** Relative expression levels of Bax, Bcl-2, Caspase-3, and cleaved Caspase-9. Data are presented as mean ± SD. **d** Western blot analysis of apoptosis-related proteins (Bax, Bcl-2, caspase-3, and cleaved caspase-9); Actin served as the loading control

To verify the impact of targeting modification on intracellular delivery and cell fate, DiO-labeled formulations were used to visualize cellular uptake. Compared with control, intracellular green fluorescence increased after carrier/drug treatments, and the RGD-HMnO₂/CDDP (G5) group exhibited the strongest DiO signal with clear spatial correspondence around nuclei, suggesting that RGD modification significantly improved internalization/retention of the nanoformulation in CT26 cells (Fig. 4a). Apoptosis was quantified by Annexin V-FITC/PI flow cytometry. Both early and late apoptosis fractions increased in treated groups relative to control, with the highest apoptotic proportion observed in the G5 group (Fig. 4b). At the molecular level, quantitative analysis of the western blot results (Fig. 4c-d) showed that the RGD-HMnO₂/CDDP (G5) group exhibited a 7-fold increase in pro-apoptotic Bax expression and a 9-fold increase in cleaved Caspase-9 levels. Conversely, the anti-apoptotic Bcl-2 expression in the G5 group was reduced to approximately 20% of the G1 levels. Notably, while the non-targeted HMnO₂/CDDP (G4) group also showed apoptotic signaling, the activation of the caspase cascade in the G5 group was significantly more robust, supporting the enhanced efficacy of the targeted delivery system. Collectively, these findings indicate that RGD-mediated targeting enhances intracellular delivery, thereby amplifying apoptosis induction by the drug-loaded nanoplatform.

### In vivo tumor accumulation, cytokine modulation, and systemic safety

**Fig. 5:**
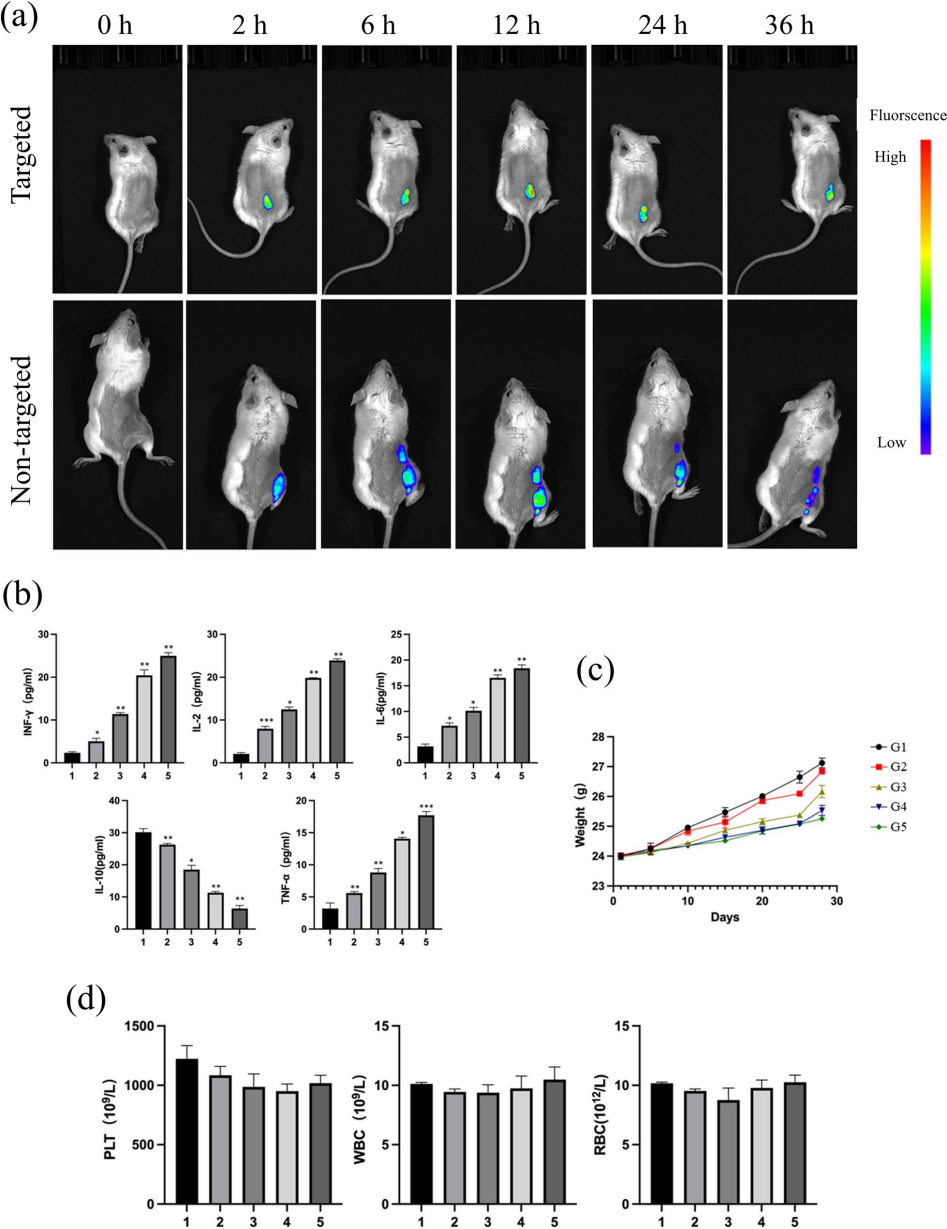
In vivo distribution, cytokine profile, and safety assessment of nanoparticle formulations in CT26 tumor-bearing BALB/c mice. **a** In vivo fluorescence imaging: CT26 tumor-bearing BALB/c mice (2 × 106cells implanted subcutaneously into the right axilla; PBS:Matrigel = 1:1; tumor volume ~100–200 mm3) received tail-vein injection of nanoparticles labeled with equal ICG doses. The targeted group (ICG–RGD–HMnO₂/CDDP) and non-targeted group (ICG–HMnO₂/CDDP) were imaged at 0, 2, 6, 12, 24, and 36 h; identical acquisition settings and a unified pseudocolor scale were used, and tumors are outlined by dashed lines (*n* = 3 per group). **b**–**d** Therapy and safety evaluation: tumor-bearing mice were randomized into PBS, HMnO₂, CDDP, HMnO₂/CDDP, and RGD–HMnO₂/CDDP groups (*n* = 5 per group) and treated via tail-vein injection at cisplatin-equivalent doses once every 3 days for four injections. **b** Serum cytokines (TNF-α, IFN-γ, IL-6, IL-2, and IL-10) measured by ELISA after the final dose. **c** Body-weight changes during treatment. **d** Endpoint blood routine indices (WBC, RBC, and PLT). Data are presented as mean ± SD. Statistical significance was assessed by one-way ANOVA with Tukey’s multiple-comparisons test; *P < 0.05, **P < 0.01, ***P < 0.001

In a CT26/BALB/c subcutaneous tumor model, in vivo fluorescence imaging was performed using equal doses of ICG-labeled formulations to assess biodistribution. Compared with the non-targeted ICG-HMnO₂/CDDP group, the targeted ICG–RGD–HMnO₂/CDDP group displayed stronger and more persistent fluorescence signals in the tumor region across 0–36 h post-injection, suggesting enhanced tumor accumulation and retention conferred by RGD modification and providing a delivery basis for therapeutic efficacy (Fig. 5a). After repeated dosing at cisplatin-equivalent levels, the RGD-HMnO₂/CDDP group exhibited more pronounced modulation of serum cytokines, including increased IFN-γ, IL-2, and TNF-α and a decreasing trend in IL-10, indicating a potential shift toward a more favorable antitumor immune milieu during treatment (Fig. 5b). Notably, body weights remained generally stable during treatment, and endpoint hematological parameters (WBC, RBC, and PLT) showed no obvious fluctuations (Fig. 5c–d), supporting overall tolerability and hematological safety within the tested dosing regimen. Taken together, these in vivo data support the feasibility and integrated advantages of RGD-HMnO₂/CDDP in terms of tumor targeting, systemic response, and safety; key drivers and mechanisms are further discussed below.

## Discussion

To address the dual challenge of inadequate delivery efficiency and systemic toxicity associated with platinum chemotherapy, we developed an RGD-modified hollow mesoporous MnO₂ nanoplatform for cisplatin delivery, integrating drug loading, active targeting, and microenvironment responsiveness. Relative to conventional passive accumulation strategies, the RGD–integrin axis provides an interpretable route for active uptake, which may improve tumor-cell internalization and local retention, thereby potentially increasing effective exposure at equivalent dose while reducing off-target tissue injury [39]. It is noteworthy that although the in vitro cytotoxicity measured by the CCK-8 assay revealed relatively comparable cell death rates between the HMnO₂/CDDP (G4) and RGD-HMnO₂/CDDP (G5) groups at certain concentrations. In the static microenvironment of a culture dish, the absence of hemodynamic shear stress and interstitial fluid pressure allows non-targeted nanoparticles (G4) to settle and interact with cells via non-specific sedimentation and diffusion pathways, which potentially masks the inherent advantages of active targeting. The true superiority of RGD modification (G5) was more accurately reflected in our in vivo investigations. Specifically, RGD-HMnO₂/CDDP demonstrated significantly enhanced and prolonged tumor accumulation compared with the non-targeted counterpart, underscoring its ability to overcome complex biological barriers that in vitro models cannot recapitulate. This design is consistent with recent advances recognizing RGD-binding integrins as actionable targets and emphasizing ligand engineering-such as tuning ligand density, spatial configuration, and multi-module integration-to enhance the robustness and reproducibility of tumor-specific delivery [39]. 

The hollow mesoporous HMnO₂ framework offers a protective reservoir for cisplatin, while MnO₂ degradation under acidic and high-GSH conditions couples drug release to hallmark features of the TME. Prior studies have shown that MnO₂-based nanosystems can deplete GSH, regulate H_2_O_2_-related reactions, and alleviate hypoxia, enabling chemical remodeling of the TME and providing a sensitization basis for chemotherapy, radiotherapy, or immunotherapy [40, 41]. In this study, ROS accumulation and mitochondrial dysfunction followed concordant patterns after treatment, suggesting that MnO₂-mediated redox perturbation may introduce an additional oxidative-stress-centered killing route beyond cisplatin-induced DNA damage.

Mechanistically, the RGD-HMnO₂/CDDP nanoplatform triggered robust ROS generation, which subsequently exacerbated oxidative stress and DNA double-strand breaks. This cascade further culminated in mitochondrial dysfunction, thereby promoting programmed cell death via the intrinsic apoptotic pathway. Given that platinum efficacy depends strongly on intratumoral concentration and intracellular exposure, the combination of nanodelivery and microenvironment modulation offers a mechanistically coherent framework for lowering resistance thresholds.

Beyond direct cytotoxicity, we observed an in vivo trend toward cytokine modulation in a pro-antitumor direction. Chemotherapy-induced immunogenic cell death (ICD) has been proposed as a bridge between intracellular stress and antitumor immune activation, involving damage-associated molecular pattern (DAMP) release, enhanced antigen presentation, and recruitment/activation of immune cells [42]. Cisplatin-induced DNA damage and MnO₂-mediated oxidative stress may jointly strengthen immune-related signaling; however, current evidence remains indirect. Future work should validate ICD-associated phenotypes by assessing calreticulin exposure, ATP/HMGB1 release, dendritic-cell maturation, and T-cell infiltration, and evaluate potential gains from combination with immunotherapy.

In terms of safety, body weight and hematological indices were largely stable during treatment, indicating acceptable tolerability within the studied dosing window. Considering potential organ toxicity associated with Mn^2+^ release, ROS generation, and platinum drugs, longer-term evaluations of renal/hepatic function, biodistribution, and histopathology are warranted, together with exposure–effect relationships matched to release kinetics. In addition, clinical translation of nanomedicines requires attention to batch-to-batch consistency, scale-up manufacturing, and critical quality attributes. Overall, this study provides mechanistically aligned evidence supporting a synergistic strategy of “targeted delivery + TME modulation” to potentiate platinum chemotherapy and informs combination-therapy design for colon cancer.

## Conclusion

The RGD-modified HMnO₂ nanocarrier loaded with cisplatin demonstrated improved anti-colon-cancer activity in vitro and in vivo. Its effects are likely attributable to enhanced cellular internalization, amplified oxidative stress and mitochondrial dysfunction, and consequent accumulation of DNA damage with activation of apoptosis-related pathways, accompanied by a tendency toward pro-antitumor modulation of immune-related cytokines. This nanoplatform provides a potential strategy and experimental basis for optimizing targeted chemotherapy delivery in colon cancer.

## Supplementary Information

Supplementary Material 1.

Supplementary Material 2.

## Data Availability

The data sets generated and/or analyzed during the current study are available from the corresponding author upon reasonable request.
