# Supplementary material for: RGD-modified hollow mesoporous nanoparticles loaded with cisplatin for antitumor therapy in colon cancer
Source: BMC Cancer. 2026 Apr 11;26:642. doi: 10.1186/s12885-026-15923-5 (PMC13188791; doi:10.1186/s12885-026-15923-5)
Supplement: Supplementary file 1 — Supplementary Material 1. [file 12885_2026_15923_MOESM1_ESM.docx]

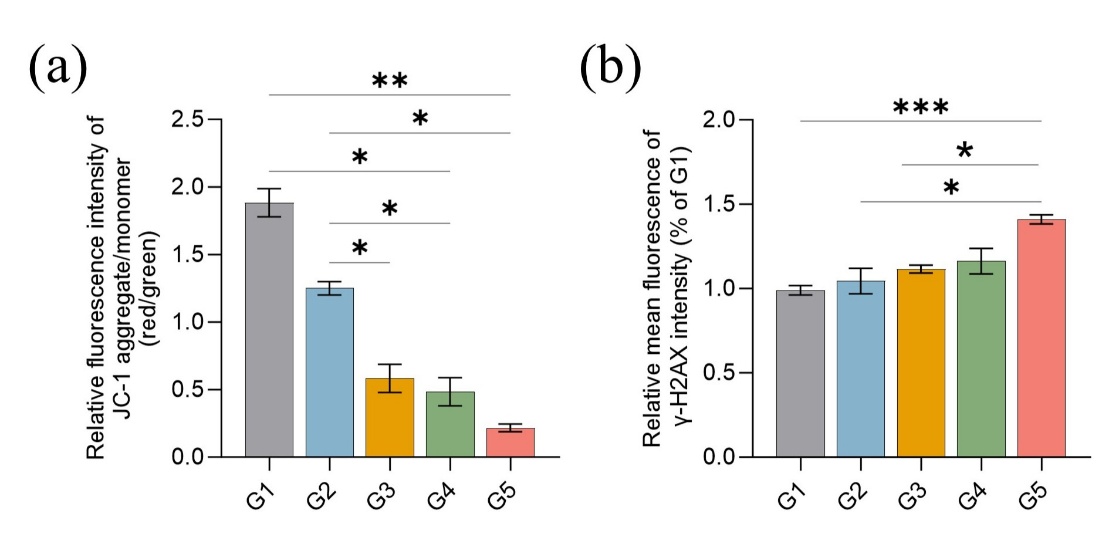
**Figure S1. Quantitative analysis of mitochondrial dysfunction, DNA damage, and apoptosis-related changes among groups.** (a) Quantification of the JC-1 assay, expressed as the red/green fluorescence ratio (JC-1 aggregate/monomer) to indicate changes in mitochondrial membrane potential. (b) Quantification of γ-H2AX immunofluorescence presented as relative mean fluorescence intensity, normalized to the G1 group. *P < 0.05, **P < 0.01, ***P < 0.001.
