# Supplementary figures and images for: RGD-modified hollow mesoporous nanoparticles loaded with cisplatin for antitumor therapy in colon cancer
Source: BMC Cancer. 2026 Apr 11;26:642. doi: 10.1186/s12885-026-15923-5 (PMC13188791; doi:10.1186/s12885-026-15923-5)

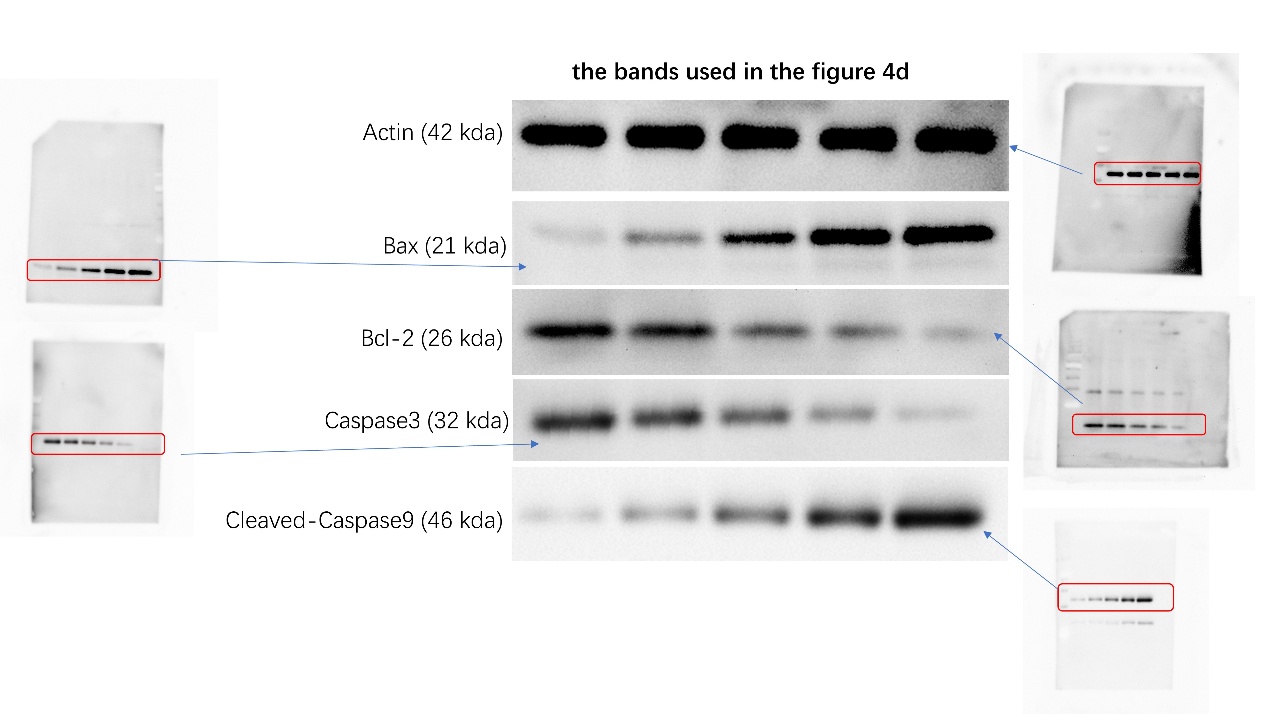


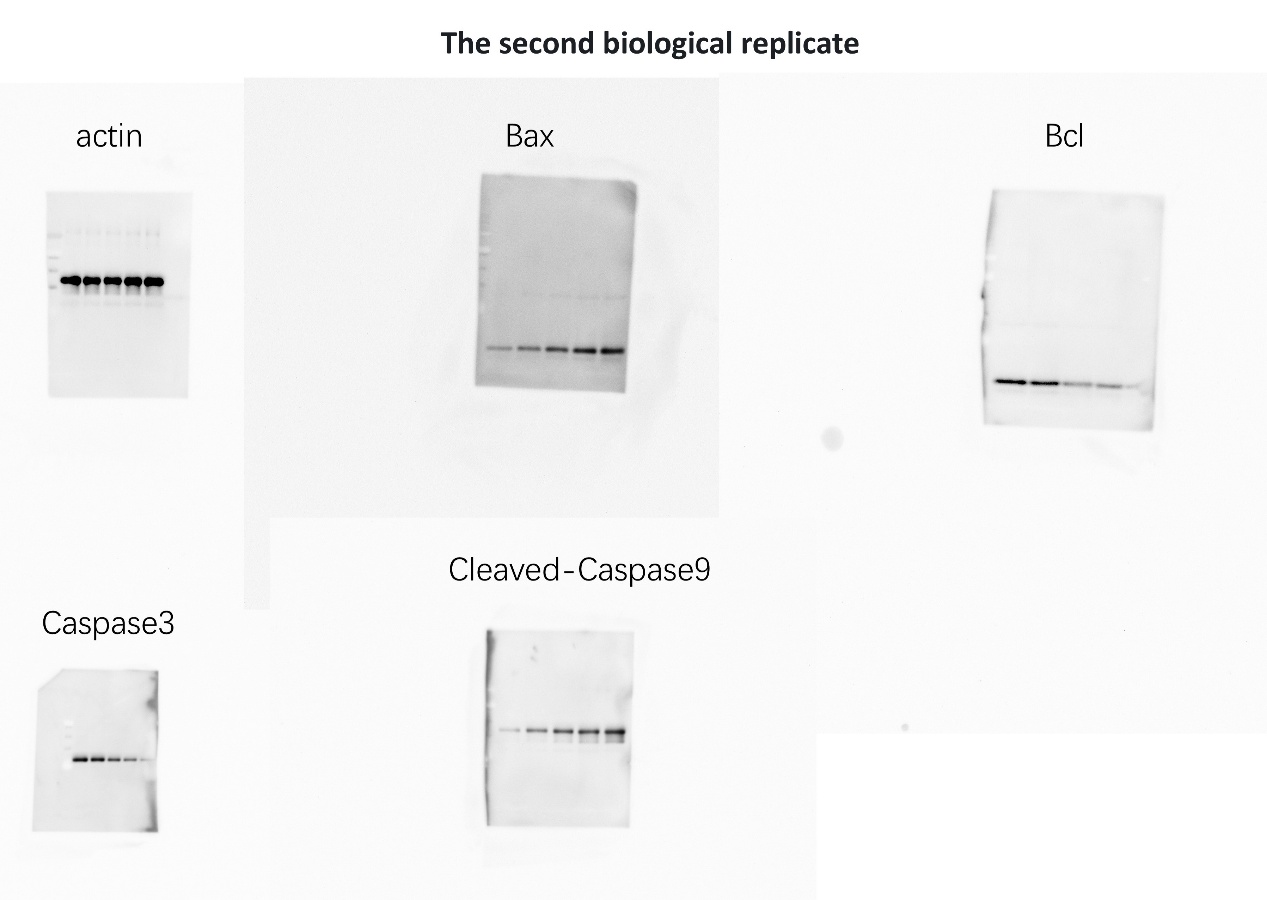


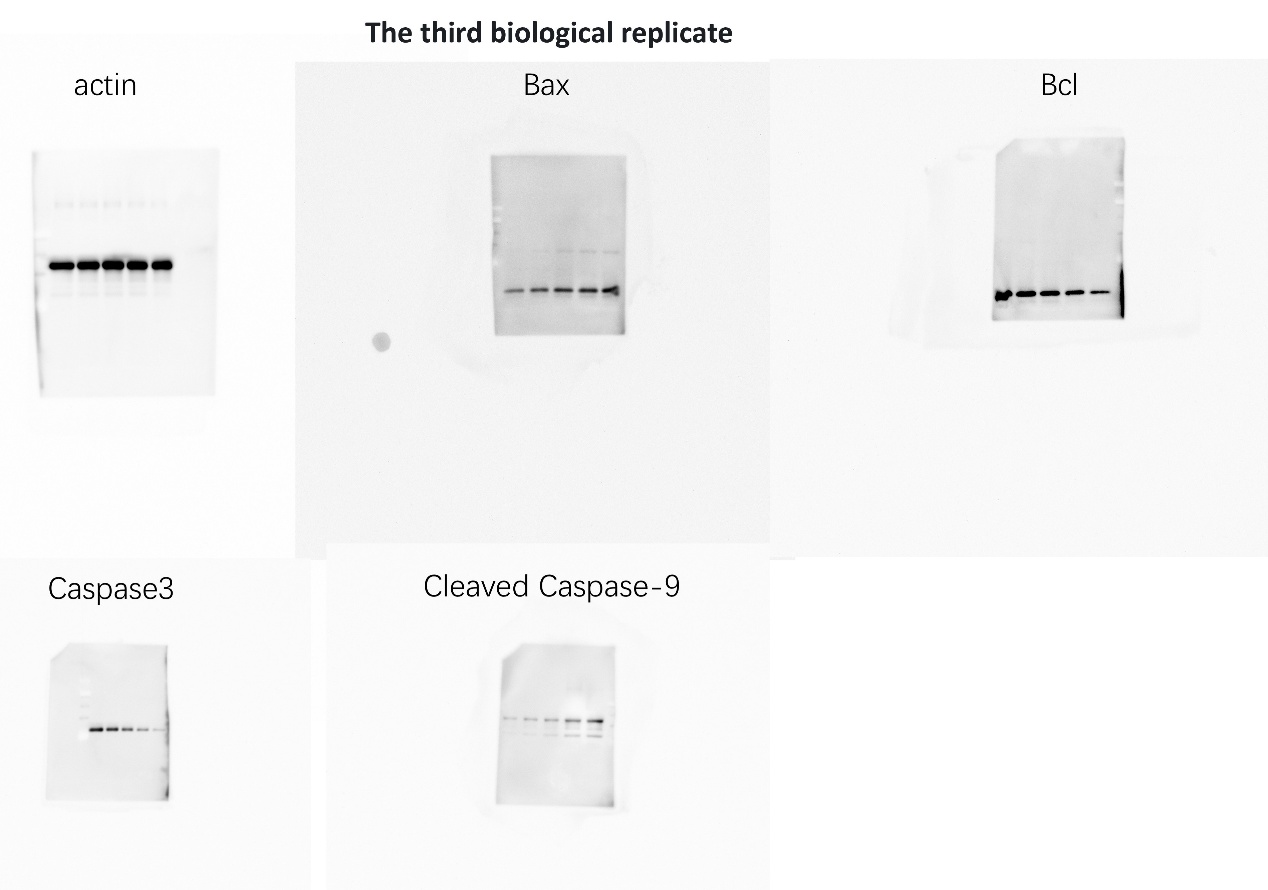

Supplement: Supplementary file 2 — Supplementary Material 2. [file 12885_2026_15923_MOESM2_ESM.docx]
